# Designing industrial work to be ‘just right’ to promote health - a study protocol for a goldilocks work intervention

**DOI:** 10.1186/s12889-022-12643-w

**Published:** 2022-02-23

**Authors:** Anders Fritz Lerche, Svend Erik Mathiassen, Charlotte Lund Rasmussen, Leon Straker, Karen Søgaard, Andreas Holtermann

**Affiliations:** 1 National Research Centre for the Working Environment, Lersø Park Allé 105, 2100 Copenhagen, Denmark; 2 Department of Sports Science and Clinical Biomechanics, University of Southern Denmark, 5230 Odense, Denmark; 3 Centre for Musculoskeletal Research, Department of Occupational Health Sciences and Psychology, University of Gävle, 80176 Gävle, Sweden; 4 Department of Public Health and Nursing, Norwegian University of Science and Technology, 7491 Trondheim, Norway; 5 Faculty of Physical Culture, Palacký University Olomouc, 77147 Olomouc, Czech Republic; 6 School of Allied Health, Curtin University, Perth, WA 6102 Australia; 7 Department of Clinical Research, University of Southern Denmark, 5230 Odense, Denmark

**Keywords:** Intervention, Goldilocks Work Principle, Health, Physical Behavior

## Abstract

**Background:**

The Goldilocks Work Principle expresses that productive work should be designed to promote workers’ health. We recently showed that it is feasible to develop and implement modifications to productive work that change physical behaviors (i.e. sitting, standing and being active) in a direction that may promote health among industrial workers. Therefore, the aim of the present study is to conduct a cluster randomised controlled trial investigating health effects of implementing the Goldilocks Work intervention among industrial workers.

**Methods:**

Our implementation plan consists of educating work teams, organizing implementation meetings, and providing feedback to workers. Three meetings with a preselected local workplace group will be scheduled. The first meeting educates the group to use a planning tool by which work can be planned to have ‘just right’ physical behaviors. The second and third meetings will focus on supporting implementation of the tool in daily work. An expected 28 clusters of work teams across two participating production sites will be randomized to either intervention or control group. Data collection will consist of 1) questionnaires regarding work and musculoskeletal health, 2) wearable sensor measurements of the physical behavior, and 3) assessment of general health indicators, including BMI, blood pressure, and fat percentage. The primary outcome is musculoskeletal health, measured by low back pain intensity, and secondary outcomes are 1) physical behaviors at work, 2) accumulated time in long bouts of sitting, standing, and being active and 3) perceived fatigue and energy during work. Furthermore, implementation and cost of the intervention will be evaluated based on questionnaires and data from the planning tool completed by the workers.

**Discussion:**

This study will evaluate the effectiveness and implementation of a 12 - weeks Goldilocks Work intervention with the aim of improving musculoskeletal health among industrial workers. The cluster randomized controlled study design and the evaluation of the implementation, results and costs of the intervention will make it capable of contributing with valuable evidence of how productive work may be designed to promote industrial workers’ health.

**Trial registration:**

Clinical trial registration was assigned 10–09-2021 (ISRCTN80969503). https://doi.org/10.1186/ISRCTN80969503

**Supplementary Information:**

The online version contains supplementary material available at 10.1186/s12889-022-12643-w.

## Introduction

### Background and rationale

Industrial workers have an increased risk for poor musculoskeletal health [1] and shortened working life expectancy [2]. This has been explained by their high physical work demands, shown to be associated with increased risk of musculoskeletal pain [1, 3] sickness absence due to pain [4, 5], and early drop-out from work [2]. Accordingly, a common approach to prevent musculoskeletal pain and fatigue among industrial workers has been to reduce their physical work demands [6, 7]. Despite these efforts, the 2018 round of the Danish Work Environment and Health Study showed that most industrial workers experience pain several days each week, and feel fatigued during work [8]. Pain and fatigue have been associated with reduced work ability [5, 9] and increased sickness absence [5, 10, 11], representing a large economic burden for organizations and society [12]. Furthermore, the current demographic projections of an aging European workforce is likely to increase the burden [13, 14]. Therefore, health promoting initiatives targeting workers with physically demanding jobs are highly warranted.

The Goldilocks Work Principle proposes that occupational physical activity can be designed to comprise a ‘just-right’ balance of physical behaviors, including intense periods, alternating with recovery, that can promote workers’ health and fitness [15]. In prior research we found that industrial workers stand for a large portion of the workday, have little variation in their physical behaviors, limited time for recovery, and limited time at an intensity which would promote cardiovascular fitness[16]. Accordingly, increasing their time spent sitting (to support recovery), their time being active, and their frequency of alternations between these behaviors could promote their musculoskeletal health [7, 15, 17]. If some of the active work time could be performed at a high intensity it might additionally benefit workers’ cardiorespiratory fitness [6].

We previously tested the feasibility of redesigning and implementing work tasks based on the Goldilocks Work Principle among a group of industrial workers in Denmark [16]. Through a participatory process, workers redesigned their work tasks with the intention of moving towards a ‘just right’ physical behavior. We set a pragmatic goal for this redesign to be, 1) an even balance of physical behaviors (i.e., equal amounts of time spent sitting, standing or active), 2) alternations between sitting, standing and active work tasks taking place about once every hour, and 3) performing high intensity work tasks for at least ten minutes every workday [16]. We showed that this intervention resulted in modest changes in physical behaviors towards the intended ‘just right’ goals. Intriguingly, workers reported lower levels of pain and fatigue after a ‘just right’ workday than after a usual workday. In order to investigate the effectiveness of ‘just right’ physical behavior to promote musculoskeletal health among industrial workers, interventions need to be tested on a larger sample using a controlled trial design whilst also monitoring the fidelity of the intervention program and the behavioral changes it introduces.

This protocol describes a cluster randomized controlled trial with the primary objective to evaluate the effectiveness of promoting musculoskeletal health by implementing changes that will move workers towards ‘just right’ physical behavior according to the Goldilocks Work Principle. A secondary objective is to determine intervention effects on behaviors measured using wearable sensors, in terms of ‘just right’ proportions and frequency of alternations between sitting, standing and active behavior during work. Finally, a third objective is to evaluate the implementation of the intervention program, including quality of the program delivery, adherence to the protocol, costs, and the extent to which the intervention was realized.

### Trial design

The Goldilocks Work intervention will use two parallel groups, with an allocation ratio of 1:1. Work teams consisting of 1–7 workers, as already organized at the workplace, will be randomly allocated to either an ‘intervention group’ receiving the intervention, or a ‘control group’ performing work as usual.

## Methods: participants, intervention and outcomes

### Study setting

A large industrial organization in Denmark was recruited for this study. Contacts and enrolment of the workplace was accomplished in collaboration with an advisory group comprising professionals from employer and employee organizations. The organization was selected because it has a large number of employees (> 200), has several production sites where workers primarily performs manual work tasks, and is motivated to participate in the project. At present (i.e., at submission), we have developed the Goldilocks Work intervention, and are conducting the implementation in the organization.

### Eligibility criteria

Inclusion criteria for participants in the recruited organization is employment for ≥ 20 h weekly. Exclusion criteria are; 1) only working night shifts, 2) known at baseline to not be employed during the entire intervention period, and 3) pregnancy.

### Development of the goldilocks work intervention

**Fig. 1:**
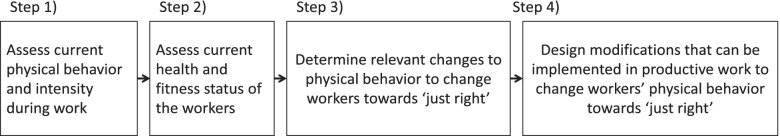
The four procedural steps recommended for developing Goldilocks Work interventions, adapted from Holtermann et al. [7]

The intervention was developed in accordance with the four-step procedure recommended for the Goldilocks Work Principle (Fig. 1) [7]. We involved stakeholders from the participating organization as well as a reference group consisting of professionals within industry to participate in the development, as recommended by Wells et al. [18].

#### Step 1 and 2

To address steps 1 and 2, we used information from the feasibility study [16] and the Danish PHysical ACTivity cohort with Objective measurements (DPhacto), containing measurements of physical behaviors [19]. Based on this information, we found that work among industrial workers typically consists of a large proportion of standing work, few alternations between sitting, standing and active work tasks, and limited time with high intensity [16, 19]. Furthermore, these workers typically exhibit a low fitness level, a high BMI (> 25 kg/cm^2^), and report pain in neck, shoulder, and lower back [19]. Based on our dialogue with stakeholders at the workplace, we found that among the workers in the present study, musculoskeletal health was the main concern. Specifically, neck, shoulder, and low back pain were identified as an issue for many workers at the participating organization.

#### Step 3

To address step 3, we established three goals to reach; 1) a ‘just right’ composition of physical behaviors (i.e., sitting, standing and active behavior) during work; 2) a ‘just right’ frequency of alternations between work tasks performed sitting, standing or active, and; 3) a ‘just right’ occurrence of periods with high intensity. Based on a collaborative process with the workplace, consisting of talks and discussions with workers, management, work environment representatives, staff representatives and health and safety representatives and with consideration to the context at this specific workplace, we formulated the following explicit ‘just right’ goals:

*The ‘just right’ composition of physical behaviors:* To get a specific estimate of the ‘just right’ composition of sitting, standing and active work we analysed data among industrial workers in the DPhacto cohort [19] to identify the composition associated with the best self-rated overall health, defined as the average composition of the 5% most ‘healthy’ workers [20]. This composition consisted in 65% sitting, 28% standing, 7% active, and was selected to represent ‘just right’ in the planned intervention. The composition is close to the recommendations of 60% sitting, 30% standing and 10% active by the European Agency for Safety and Health at Work [21], and for communicative purposes at the workplace, we used this composition of 60%/30%/10%. Everyone in the collaborative process agreed that this composition was a feasible and relevant goal to aim for.

*The ‘just right’ alternations between sitting, standing and active work tasks:* To determine the ‘just right’ frequency of alternations, we discussed the feasibility of alternating between work tasks during productive work with workers and management at the workplace. The current evidence supporting physical variation to have a protective effect against development of musculoskeletal disorders in working life is not conclusive [22–24]. In our previous study, we observed that workers alternated once during usual work and three times during modified work between sitting, standing and active work tasks. This increase in alternations may have contributed to the observed reduction in pain and fatigue following modified work [16]. Discussions with workers and management at the current workplace revealed limitations in the options for alternating between tasks. At first, we suggested that workers should change task every 30 min, with the intention of limiting pain following prolonged periods of standing [25, 26]. However, management expressed concerns about negative effects on productivity, i.e., that passing on work tasks from one worker to another would occupy a substantial part of the work time. Further, when facing a change every 30 min, workers expressed concerns about never experiencing the satisfaction of completing a work task. Eventually, these discussions resulted in an agreement that changing work tasks every 60 min would be feasible without compromising productivity or affecting workers’ perceptions, while still being a relevant goal to aim for.

*The ‘just right’ periods with high intensity:* Even small amounts of high intensity physical activity can have beneficial health effects [27]. However, explicitly aiming for more time at a high intensity physical activity among industrial workers may neither be feasible nor beneficial, if it, for example, occurs without sufficient time for recovery. When discussing opportunities to perform high intensity physical activity as a part of productive work with the participating workplace, we identified several challenges. The most important challenges were, 1) concern by management and health & safety representative about an increased risk of accidents during production as a consequence of performing work with high intensity; 2) struggles by workers and work environment representatives to find a way to safely introduce periods with high intensity in their current work tasks; 3) concern by the staff representatives about resistance from workers to work with high intensity, since some workers might perceive that as an attempt by management to increase their productivity; 4) concern by the health & safety representative and management that implementing high intensity work tasks would counteract their current safety focus to stay safe by ‘never rushing’. During our discussions, we made extensive efforts to accommodate all these challenges in the intervention. Despite our efforts, we were unable to come up with satisfying solutions, and did not reach any agreement of a feasible and potential beneficial goal to aim for. Consequently, we decided to omit the goal of including periods with high intensity in the ‘just right’ work design from the current intervention.

#### Step 4

We designed a general planning tool (i.e., a modification that could likely be implemented, step 4 in Fig. 1) in Excel (Microsoft Office Professional Plus 2016) with the purpose of providing workers with a specific instrument by which they could organize their work tasks. The purpose of this tool is to have workers modify their jobs towards the ‘just right’ physical behavior. To make the tool implementable and applicable for workers, it will be tailored to their work context. Prior to the intervention, a member from our research team will involve the work environment representatives to identify all relevant work tasks, and then have them determine the typical physical behavior for each task as either sitting, standing or active. To ensure that the tool properly reflects the work context at any time, work tasks can be added or modified (e.g. by changing their predefined physical behavior) during the intervention, based on feedback from workers and the local workplace group. At any time, the planning tool includes the existing work tasks, so that workers can select them from a drop-down list, and plan their working day. In the present study, the planning tool partitions the workday into six blocks of approximately one hour each. The objective for each work team of 1 to 7 workers is to rearrange and swap work tasks in production on a given day until everyone has the best possible ‘Goldilocks index’. The ‘Goldilocks index’ will be generated by the general tool, ranging between 0 and 100 (where 100 is the best score possible) and based on how close the plan is to being ‘just right’. To guide the workers, the ‘Goldilocks index’ is color-coded: red if the index is < 50 points, yellow if the index is 50–79, or green if the index is ≥ 80.

**Fig. 2:**
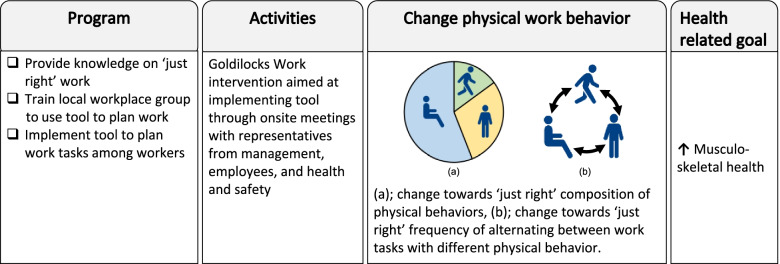
Illustration of the program logic for the intervention developed in collaboration with stakeholders from the participating organization as a result of the 4-step model of the Goldilocks Work Principle.

**Fig. 3:**
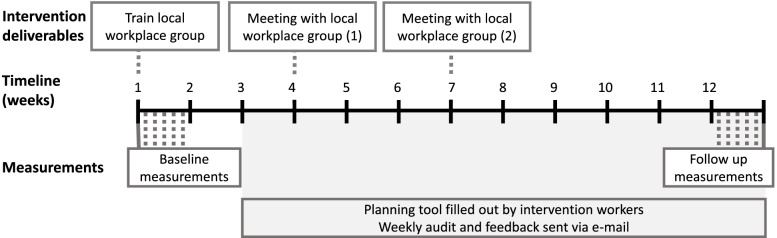
Timeline of the 12-week intervention period.

We summarized the decisions made in the 4-step process in a program logic (Fig. 2). The program logic is a visual representation of the intended changes in the intervention program and activities, how they are expected to change physical work behavior, and how that will, eventually, improve the health-related goal [28]. The program logic was used as a simple illustration to share with stakeholders [28] and to guide the planning of the intervention, deciding when to do what, and with whom (Fig. 3).

### Implementation plan for the goldilocks work intervention

Because the intervention is conducted at the workplace, fidelity towards the implementation of the intervention program is crucial when assessing its effectiveness [29]. Fidelity is defined as the degree to which an intervention is implemented as prescribed in the original protocol, or as intended by the program developers [30]. Thus, the intervention program must be implemented with fidelity in order for the desired behavior changes to happen and eventually result in the expected health outcome, as visualized in the program logic (Fig. 2) [31].

We selected four strategies to successfully implement the intervention program (Table 1). The main considerations when selecting these strategies were the required time resources, the feasibility of conducting the strategies, and previous experience from within the local workplace group regarding implementation of new initiatives in the organization. Additionally, as a part of the development of the planning tool, we defined four key variables that were monitored by the planning tool and fed back to the workers during the intervention (Table 1, audit and feedback strategy). These variables were chosen based on the assumption that they would reveal relevant information about fidelity, such as adherence and actual use of the planning tool [30, 31]. The four variables are; 1) the extent to which workers make work plans using the planning tool; 2) the amount of time workers use to fill out the planning tool; 3) the amount of red, yellow and green workdays that workers plan, and; 4) the extent to which workers can adhere to their plan.

**Table 1:** Implementation plan (cf. Proctor et al., [32])

Name of strategy	Definition of strategy	The actors	The action	The target	Temporality	Dose	Implemen-tation outcome affected
Inform about project	Inform about the project aim and requirements for participation	Researchers and team leaders	Present project at weekly status meetings and distribute folders on project and data security	Disseminate knowledge and address questions about wearing sensors and data protection	Before intervention	Three 20 min meetings	Fidelity and participation rate
Conduct educational meeting [33]	Engage stakeholders in disseminating knowledge about planning tool to workers in production	Researchers and local workplace group	Train work environment representatives to use planning tool and select which workers they are responsible for training	Involve and motivate staff to take ownership of the implementation of the intervention	During intervention	One 1.5 h meeting	Fidelity
Audit and feedback [34]	Motivate workers to change organization of their work tasks	Researchers and workers	Provide weekly feedback on four key variables related to the planning tool	Motivate workers to change their physical behavior	During intervention	Weekly e-mails	Fidelity
Organize implementation team meetings [33]	Discuss implementation effort, share lessons learned, and support one another in the group	Researcher and local workplace group	Discuss how intervention should move forward and which initiatives should be prioritized	Involve management and employees to take ownership of the implementation of the intervention	During intervention	Two 1.5 h meetings	Fidelity

Prior to the intervention, we will form a local workplace group at each production site consisting of work environment representatives, and representatives from management, staff and the health & safety department. In organizing this group we will involve stakeholders with the intention of making the process participatory [18], and support implementation fidelity. During the intervention, we will schedule one educational meeting and two implementation meetings inviting the local workplace group to participate (Fig. 3).

The first meeting (i.e., ‘train local workplace group’, Fig. 3) consists of a researcher training/educating the local workplace group to use the tool for planning work tasks. The local workplace group and the researchers then arrange how, when and who should train the workers to use the tool.

The second and third meeting (i.e., ‘meeting with local workplace group’ 1 & 2, Fig. 3) focus on the use of the tool for work planning. During these meetings, researchers will feed information about the implementation and the fidelity back to the local workplace group [29]. Researchers will present and discuss the four key variables in the planning tool as well as inputs from the workers (verbally or via e-mail). Based on this information, the local workplace group will determine relevant initiatives to support implementation of the intervention. (Table 1, organize implementation team meetings).

### Outcomes

#### Primary outcome measure

The primary outcome measure for the trial will be the difference between intervention and control groups in the change of low back pain intensity from baseline to follow up (12 weeks). We chose low back pain as an overall representation for musculoskeletal health (Fig. 2.), since it has a pronounced influence on both work ability [9], sick leave [10], and since it occurs in many pain patterns [35]. Pain intensity will be measured repeatedly on a numeric scale (NRS, 0–10). A question will be sent to the participants in a text message at the end of the workday for five consecutive workdays at baseline (week 1) and five consecutive workdays at follow up (week 12), asking them to rate their low back pain intensity.

#### Secondary outcome measures

Secondary outcomes include:

- Difference between intervention and control group in the change in physical behavior composition (i.e., sitting, standing and active), measured using accelerometry for five workdays at baseline and five workdays at follow up.
- Difference between intervention and control group in the change in accumulated time in bouts of sitting, standing and active, measured using accelerometry for five workdays at baseline and five workdays at follow up. Bout length will be defined as either short (< 5 min), medium (5-30 min), or long (> 30 min).
- Differences between intervention and control group in the change in perceived fatigue and energy level, perceived physical exertion, and self-rated productivity measured using a numeric scale from 0–10 for five workdays at baseline and five workdays at follow up.
- Adherence to the intervention program measured through participation rates at planned meetings, and records of planned *and* conducted activities during the intervention.
- Use of the planning tool measured through the number of completed planning tool schedules.
- Quality of the program delivery measured using questionnaires following training and implementation meetings with the local workplace group.
- Cost-effectiveness of the intervention, i.e. the ratio of incremental cost and incremental change in low back pain intensity.

### Participant timeline

The schedule of enrolment, intervention and assessment is presented in Table 2. Together with the organization, we found suitable and interested production sites. Within these participating production sites we will randomize clusters (i.e., teams of workers), to either intervention or control. Subsequently, workers within each cluster will be invited to participate, assessed for eligibility, and followed during the intervention period. Non-participants, loss to follow up, the extent of lost data and the amount of data used for final analysis will be reported.

**Table 2:**
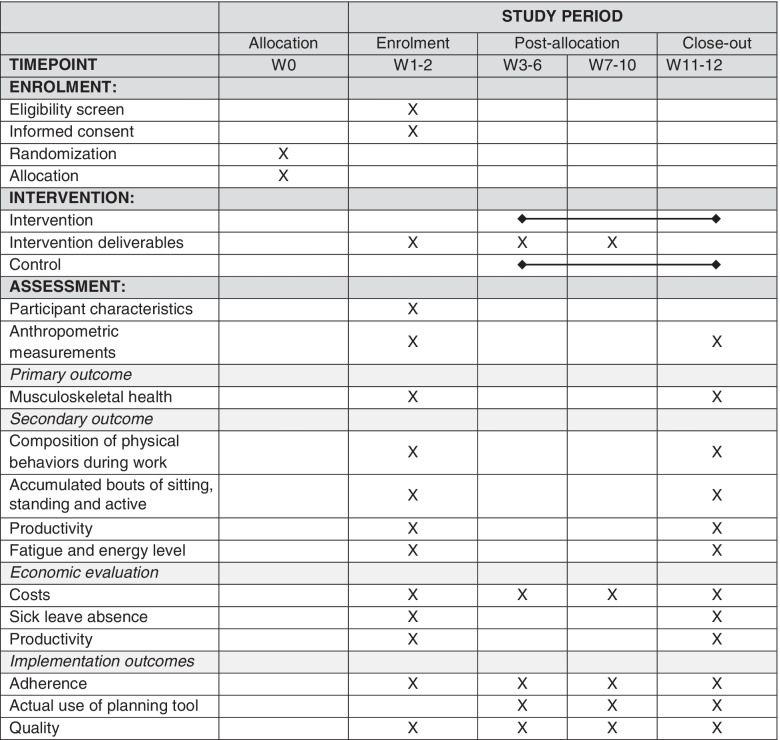
Schedule of enrolment, intervention, and assessments (cf. Chan et. al., [36])

### Sample size

Previously, we have experienced a participation rate among eligible industrial workers of 84%, followed by a response rate of 87% of those participating [37]. At present, management in the participating organization has estimated that 110 workers will be eligible for the present study. Based on our previous experience, we assume that 80% will be willing to participate in our study (i.e., 88 workers) and that 20% will be lost to follow up. Thus, assuming that 70 participants, organized in clusters with an average size of *n* = 4 (range *n* = 2 to *n* = 7), will complete the study, we may estimate the detectable effect size in a cluster randomized controlled design as follows:

In our previous feasibility study among industrial workers [16], we found that low back pain intensity was, on average, 3.7 points (on a 0–10 scale) with a total variance between workers of 10.6. This total variance will be reduced with repeated measurements over days in each subject. Within-subject (between-days) variance was 1.6, or 15% of the total variance (between-subject and within-subject) of low back pain. If we repeat measurements for 5 days, the within-subject variance will be reduced to 0.3, resulting in an estimated total variance of 9.3. Based on this, we can – given the sample size stated above, i.e. *n* = 70 – expect to be able to detect a 1.5 point difference between groups in low back pain intensity, at a significance level of 0.05, a power of 0.80, and a cluster size of 4 (the average size).

The effect on low back pain intensity from redesigning physical behaviors at work is unknown. In our feasibility study, we found a mean difference of 1.1 in low back pain intensity between usual and redesigned workdays [16]. However, we cannot draw any firm conclusion about the expected effect size from that study due to the difference in study design, the limited sample size (*n* = 14) and, hence, the large uncertainty in data. Nevertheless, reducing low back pain intensity by 1.5 is likely to be a meaningful clinical and work-related change [38]. Therefore, we find it meaningful to conduct the present study despite its uncertainties.

## Methods: assignment of interventions

First, workers will be asked by a researcher if they wish to participate in the study. Workers agreeing will then sign an informed consent, and workers declining will be counted as non-participants. After signing the informed consent, workers will complete a physical assessment and following that, they will be verbally informed about their group allocation. This is necessary because we need to plan training activities with the work teams and the local workplace group. Additionally, we need to prepare the planning tool to comply with the specific work tasks of the work teams allocated to the intervention.

Due to the nature of the intervention, it is not possible to blind the workers to their allocation. Additionally, during enrolment, blinding of the assessors is not possible. However, the main outcome, musculoskeletal health, is measured through questionnaires sent out via text messages, and thus, is not affected by our inability to blind the assessors during enrolment. Furthermore, all secondary outcomes are collected with either wearable sensors or questionnaires, and will not be biased by lack of assessor blinding.

Clusters (i.e. teams) will be randomly allocated to the intervention and control groups in a 1:1 ratio. The teams will be allocated using SAS version 9.4 and the function RANUNI, which returns a number generated from a uniform distribution on the interval (0, 1) [39]. The randomization process will be performed separately for each production site, i.e., the randomization will be stratified. Clusters within a production site receiving values in the lower half of numbers drawn at that site will be assigned to the intervention, and clusters with values in the upper half will be controls. A data manager not otherwise involved in conducting the intervention will generate the allocation sequence for each production site.

Intervention and control workers will be present at all included production sites. This allocation procedure was chosen to reduce the risk of bias due to changes in contextual factors (e.g., change in management, structural changes, or supply chain challenges) during the intervention period, which have been shown to occur quite often [40]. Furthermore, it may also reduce potential bias from contextual differences that may exist between sites, such as differences in work tasks, restrictions in production complicating work planning, or how machines are operated.

## Methods: data collection, management and analysis

### Data collection methods

Data will be collected for all workers at baseline (week 1) and follow up (week 12). Data from intervention workers’ planning tools will be collected every day from week 3 to 12. Records of participation rates (i.e., in educational, and implementation team meetings, cf. Table 1), and costs related to the intervention (e.g., staff time and consultant time) will be collected throughout the intervention period. All sensitive personally identifiable information will be collected at the workplace by the researchers. The information will then be transported back to the research center and will be uploaded on a secure drive, which is accessible only to researchers physically present at the center. Finally, when all information has been collected, one single file for each participant will be stored in a secure place. These files will remain stored for a period of 3 years after completion of the study. Once the dataset is complete, the information uploaded to the secure drive will be anonymized by a data manager.

### Participant characteristics and anthropometric measurements.

We will collect information about sociodemographic factors including age, gender, ethnicity, length of service, job title, and weekly work hours. Further, we will collect information about smoking and alcohol consumption [41]. We will measure height (Seca 213; Seca GmbH, Hamburg, Germany), weight (BC-418 MA body composition analyzer; Tanita, Tokyo, Japan), and calculate the body mass index (body weight [kg]/(body height squared [m2])). In addition, we will determine fat percentage (DC-430 SMA body composition analyzer; Tanita, Tokyo, Japan) and resting blood pressure (Omron M3 or Omron M6 Comfort; Omron Corporation, Kyoto, Japan) using standardized protocols.

### Physical behaviors

The protocol for measuring physical behaviors will be similar to that used in previous studies [16, 19, 42]. An AX3 accelerometer (3-Axis Logging Accelerometer; Axivity Ltd., Newcastle upon Tyne, UK) will be mounted using adhesive tape (Hair-Set double-sided adhesive tape; 3 M Company, Maplewood, MN, USA) on the worker’s right thigh at the most muscular part of the quadriceps femoris, midway on the line between the anterior inferior iliac spine and the top of the patella. Workers will be asked to wear the accelerometer around the clock for five consecutive days at baseline and follow up, and will be instructed on how to change the adhesive tape if necessary. At the end of day five, the accelerometer will be returned to the research team. Data will be downloaded using the manufacturer’s software (OMGUI, Configuration and Analysis Tool). A workday will be excluded from further analysis if accelerometer recordings are available for less than 4 h. Periods > 60 min without movement will be regarded as non-wear time or sleep.

Physical behaviors will be exhaustively classified using the Acti4 software [42] into time spent sitting or lying (termed sitting below), standing (consisting of time spent standing still, or standing with slight movement), and active (consisting of time spent walking, running, stair climbing or cycling).

During all measurement days, the workers will note in a diary at what time they: 1) woke up, 2) arrived at work, 3) left work, 4) went to sleep, and 5) if the accelerometer was detached at any time during data collection. On the basis of the diaries, the continuous timeline of physical behavior data will be partitioned into periods of, 1) work, 2) leisure time awake, and 3) time in bed.

### Planning tool

All workers in the intervention group will receive an e-mail immediately after baseline measurements with a unique link to an online folder containing the Excel planning tool. There will be a planning tool sheet every workday for the entire 10 weeks of the intervention (i.e., week 3 to 12, Fig. 3). Data will be automatically stored on an online drive (Microsoft, One Drive, 2021). Eventual data containing information about the four key variables evaluating fidelity will be saved on the secure drive.

### Questionnaires

The questionnaires consist of standard validated questions, as well as questions to describe the intervention process. At baseline and follow up we will collect information about musculoskeletal health [43], general health [44], need for recovery [45], self-assessed physical fitness [46], sickness absence, psychosocial work environment [47] and productivity [48–50]. Questionnaires using a numerical scale from 0–10 will be send at the end of the workday, for each of the first five days and the last five days of the intervention on; 1) pain, 2) fatigue and 3) energy [43]. Furthermore, customary questions will be used to collect information about participants’ use of medication and healthcare services.

### Quality of program delivery

Specific questions to evaluate the fidelity towards the intervention program will be formulated to reflect program quality, and motivation towards the intervention. These will be sent out to the local workplace group and course leaders using text messages after completion of all the training and implementation meetings. Furthermore, notes and records from meetings with the local workplace group will be used to assess fidelity regarding the implementation of the intervention.

### Costs

Costs of conducting the intervention activities will be collected. Costs include resources used by the organization to implement the intervention, and resources used by the researchers to deliver the intervention. Implementation costs for the organization will include staff time used by workers, work environment representatives, health & safety advisors and managers participating in training and implementation meetings. Time used by staff for other activities related to implementing the intervention will also be included. Costs will be estimated on basis of the average gross salaries of staff, including overhead. Costs of delivering the intervention for researchers will include the time used for preparing and conducting the training and implementation meetings, and any additional planning and meeting related to implementation of the intervention. Costs for researchers will be estimated based on their hourly fee, including overhead. Finally, costs related to workers’ productivity, sickness absence and consumption of medicine and health services (e.g., visits to a general practitioner or a physical therapist) will be collected at baseline and follow up.

### Statistical methods

Primary outcome will be assessed as the difference between intervention and control groups in change in low back pain following the 12 weeks intervention. We will use both intention-to-treat effect estimates and per-protocol effect estimates to assess the effectiveness of the intervention, as recommended when evaluating trials conducted in real life settings [51]. The per-protocol criteria is defined as work teams (clusters) who completes ≥ 60% of the available planning tools. Based on the distribution of data, parametric or non-parametric test will be performed.

The secondary outcomes include data of different types, i.e. categorical, continuous and compositional data. For all data types, descriptive statistics relevant to the variable will be applied. The statistical analysis used to assess differences between intervention and control groups will also be adapted to the type and distribution of the data. Specifically, changes in the physical behavior composition (i.e. sitting, standing, and active behavior), will be analyzed using CoDA metrics.

A detailed analysis plan describing comparison and test of changes in the intervention and control groups will be completed before initiating the analysis and will be available upon reasonable request.

## Discussion

This protocol describes a 12-weeks Goldilocks Work intervention among industrial workers. The primary hypothesis is that implementing a ‘just right’ physical behavior among workers will lead to improved musculoskeletal health.

### Strengths and limitations

The 4-step procedure and participatory approach that guided the development of the program logic and the intervention is a strength of this protocol. The cluster randomized controlled design with randomization performed at each production site is a strength because it reduces potential bias. The planning tool adapted to the workers’ context is a strength because it offers an easy-to-use and practical approach for their own planning of work tasks to change behavior. The approach of feeding back information from the planning tool to the workers is also a strength that can improve the likelihood that workers’ behavior is changed [34]. Furthermore, the information collected about the implementation process is a strength because it may inform details about who did what when, and explain why things turned out as they did.

A limitation is that participants are not blinded to their allocation; this can increase the risk of selection bias. However, workers will not receive knowledge of their allocation until after they completed their eligibility screen and health check. Another limitation is the short duration of the study (i.e., 12 weeks), since it may take longer to effectively implement changes in physical behavior and thus effectively improve musculoskeletal health. However, even if the intervention is not effective, the workers and the workplace will gain valuable knowledge about their work context, the current physical behavior, and which changes that may improve workers’ health on the long term.

## Supplementary Information

**Additional file 1: Appendices.** Written informed consent form.

## Data Availability

Data sharing is not applicable to this article as no datasets were generated during the current study. Datasets generated and/or analysed following this protocol will not be publicly available because they will contain sensitive personally identifiable information, but they will be available from the corresponding author on reasonable request. We plan to communicate our results from any study conducted on the basis of this study protocol through publication of scientific articles and through a report of the results to the Danish Working Environment Research Fund. Furthermore, the results of the study will be communicated to relevant stakeholders (e.g., workers, management, work environment department) in the participating organization. In the case that important protocol modifications are made (e.g. changes to eligibility criteria, outcomes, or analyses) we will inform all relevant stakeholders (e.g., researchers, trial registries) about the modifications, including explaining the reason for any modification made.

## Abbreviations

DPhacto: Danish PHysical ACTivity cohort with Objective measurements
SPIRIT: Standard Protocol Items Recommendations for Interventional Trials
CONSORT: CONsolidated Standards Of Reporting Trials

## References

[1] Andersen LL, Fallentin N, Thorsen SV, Holtermann A (2016). Physical workload and risk of long-term sickness absence in the general working population and among blue-collar workers: prospective cohort study with register follow-up. Occup Environ Med.

[2] Pedersen J, Schultz BB, Madsen IEH, Solovieva S, Andersen LL. High physical work demands and working life expectancy in Denmark. Occup Environ Med. 2020;77:576–82. 10.1136/oemed-2019-106359 PMC7402449 32398291

[3] Andersen LL, Vinstrup J, Sundstrup E, Skovlund SV, Villadsen E, Thorsen SV (2021). Combined ergonomic exposures and development of musculoskeletal pain in the general working population: A prospective cohort study. Scand J Work Environ Health.

[4] Holtermann A, Hansen JV, Burr H, Søgaard K (2010). Prognostic factors for long-term sickness absence among employees with neck-shoulder and low-back pain. Scand J Work Environ Health.

[5] Hallman DM, Holtermann A, Dencker-Larsen S, Birk Jørgensen M, Nørregaard Rasmussen CD. Are trajectories of neck–shoulder pain associated with sick leave and work ability in workers? A 1-year prospective study. BMJ Open. 2019;9(3):e022006. 10.1136/bmjopen-2018-022006. 10.1136/bmjopen-2018-022006 PMC6475446 30898794

[6] Straker L, Mathiassen SE (2009). Increased physical work loads in modern work–a necessity for better health and performance?. Ergonomics.

[7] Holtermann A, Mathiassen SE, Straker L (2019). Promoting health and physical capacity during productive work: the Goldilocks Principle. Scand J Work Environ Health.

[8] Work environment and health in Denmark, 2012-2018: The National Research Centre for the Working Environment; 2020 [cited 2020 21/06]. Available from: https://arbejdsmiljodata.nfa.dk/.

[9] Skovlund SV, Bláfoss R, Sundstrup E, Andersen LL (2020). Association between physical work demands and work ability in workers with musculoskeletal pain: cross-sectional study. BMC Musculoskelet Disord.

[10] Lötters F, Burdorf A (2006). Prognostic factors for duration of sickness absence due to musculoskeletal disorders. Clin J Pain.

[11] Janssen N, Kant IJ, Swaen GM, Janssen PP, Schröer CA. Fatigue as a predictor of sickness absence: results from the Maastricht cohort study on fatigue at work. Occup Environ Med. 2003;60:i71–i76. 10.1136/oem.60.suppl_1.i71 PMC1765725 12782750

[12] Fatoye F. The economic impact of musculoskeletal pain. The Journal of Physiotherapy Pain Association. 2018;2018:3–4(2).

[13] Demographic trends of workforce: European Commission; [cited 2020 14/12]. Available from: https://knowledge4policy.ec.europa.eu/foresight/topic/changing-nature-work/demographic-trends-of-workforce_en.

[14] Andersen LL, Pedersen J, Sundstrup E, Thorsen SV, Rugulies R. High physical work demands have worse consequences for older workers: prospective study of long-term sickness absence among 69 117 employees. Occup Environ Med. 2021;78:829–34. 10.1136/oemed-2020-107281 PMC8526881 33972376

[15] Straker L, Mathiassen SE, Holtermann A. The “Goldilocks Principle”: designing physical activity at work to be “just right” for promoting health. Br J Sports Med. 2018;52(13):818–9. 10.1136/bjsports-2017-097765 PMC6029635 28663212

[16] Lerche AF, Mathiassen SE, Rasmussen CL, Straker L, Søgaard K, Holtermann A. Development and Implementation of 'Just Right' Physical Behavior in Industrial Work Based on the Goldilocks Work Principle-A Feasibility Study. Int J Environ Res Public Health. 2021;18(9):4707. 10.1371/journal.pone.0245501. 10.3390/ijerph18094707 PMC8125316 33925078

[17] Øverås CK, Villumsen M, Axén I, Cabrita M, Leboeuf-Yde C, Hartvigsen J (2020). Association between objectively measured physical behaviour and neck- and/or low back pain: A systematic review. Eur J Pain.

[18] Wells R, Norman R, Frazer M, Laing A, Cole D, Kerr M (2003). Participative Ergonomic Blueprint.

[19] Jørgensen MB, Gupta N, Korshøj M, Lagersted-Olsen J, Villumsen M, Mortensen OS (2019). The DPhacto cohort: An overview of technically measured physical activity at work and leisure in blue-collar sectors for practitioners and researchers. Appl Ergon.

[20] Dumuid D, Wake M, Burgner D, Tremblay MS, Okely AD, Edwards B, et al. Balancing time use for children’s fitness and adiposity: Evidence to inform 24-hour guidelines for sleep, sedentary time and physical activity. PLOS ONE. 2021;16(1):e0245501. 10.1371/journal.pone.0245501. PMID: 33465128; PMCID: PMC7815105. 10.1371/journal.pone.0245501 PMC7815105 33465128

[21] Peereboom K, de Langen N, Bortkiewicz A, Copsey S. Prolonged constrained standing at work - executive summary. European Agency for Safety and Health at Work; 2021. https://www.google.com/url?sa=t&rct=j&q=&esrc=s&source=web&cd=&cad=rja&uact=8&ved=2ahUKEwj1gbDW4OP1AhVQasAKHeeNC68QFnoECBEQAQ&url=https%3A%2F%2Fhealthy-workplaces.eu%2Fsites%2Fdefault%2Ffiles%2Fpublications%2Fdocuments%2FStandingatworksummaryEN.pdf&usg=AOvVaw2xCmAaNz6Tpo38UDU50X4z.

[22] Mathiassen SE (2006). Diversity and variation in biomechanical exposure: what is it, and why would we like to know?. Appl Ergon.

[23] Leider PC, Boschman JS, Frings-Dresen MH, van der Molen HF (2015). Effects of job rotation on musculoskeletal complaints and related work exposures: a systematic literature review. Ergonomics.

[24] Dennerlein JT, Evangelista GDS, Rodrigues da Silva P, Padula RS, Comper MLC (2017). Effectiveness of job rotation for preventing work-related musculoskeletal diseases: a cluster randomised controlled trial. Occup Environ Med..

[25] Coenen P, Parry S, Willenberg L, Shi JW, Romero L, Blackwood DM (2017). Associations of prolonged standing with musculoskeletal symptoms-A systematic review of laboratory studies. Gait Posture.

[26] Callaghan JP, De Carvalho D, Gallagher K, Karakolis T, Nelson-Wong E (2015). Is Standing the Solution to Sedentary Office Work?. Ergonomics in Design.

[27] Ekelund U, Tarp J, Fagerland MW, Johannessen JS, Hansen BH, Jefferis BJ (2020). Joint associations of accelerometer-measured physical activity and sedentary time with all-cause mortality: a harmonised meta-analysis in more than 44 000 middle-aged and older individuals. Br J Sports Med.

[28] Mills T, Lawton R, Sheard L (2019). Advancing complexity science in healthcare research: the logic of logic models. BMC Med Res Methodol.

[29] Bauer MS, Damschroder L, Hagedorn H, Smith J, Kilbourne AM (2015). An introduction to implementation science for the non-specialist. BMC Psychology.

[30] Proctor E, Silmere H, Raghavan R, Hovmand P, Aarons G, Bunger A (2011). Outcomes for implementation research: conceptual distinctions, measurement challenges, and research agenda. Adm Policy Ment Health.

[31] Mihalic S (2004). The importance of implementation fidelity. Emotional and Behavioral Disorders in Youth.

[32] Proctor EK, Powell BJ, McMillen JC (2013). Implementation strategies: recommendations for specifying and reporting. Implement Sci.

[33] Powell BJ, Waltz TJ, Chinman MJ, Damschroder LJ, Smith JL, Matthieu MM (2015). A refined compilation of implementation strategies: results from the Expert Recommendations for Implementing Change (ERIC) project. Implement Sci.

[34] Ivers NM, Sales A, Colquhoun H, Michie S, Foy R, Francis JJ (2014). No more ‘business as usual’ with audit and feedback interventions: towards an agenda for a reinvigorated intervention. Implement Sci.

[35] Hartvigsen J, Davidsen M, Hestbaek L, Sogaard K, Roos EM (2013). Patterns of musculoskeletal pain in the population: A latent class analysis using a nationally representative interviewer-based survey of 4817 Danes. Eur J Pain.

[36] Chan AW, Tetzlaff JM, Altman DG, Laupacis A, Gøtzsche PC, Krleža-Jerić K (2013). SPIRIT 2013 statement: defining standard protocol items for clinical trials. Ann Intern Med.

[37] Gupta N, Wåhlin-Jacobsen CD, Abildgaard JS, Henriksen LN, Nielsen K, Holtermann A (2018). Effectiveness of a participatory physical and psychosocial intervention to balance the demands and resources of industrial workers: A cluster-randomized controlled trial. J Scandinavian Journal of Work, Environment Health.

[38] Hawker GA, Mian S, Kendzerska T, French M. Measures of adult pain: Visual Analog Scale for Pain (VAS Pain), Numeric Rating Scale for Pain (NRS Pain), McGill Pain Questionnaire (MPQ), Short-Form McGill Pain Questionnaire (SF-MPQ), Chronic Pain Grade Scale (CPGS), Short Form-36 Bodily Pain Scale (SF-36 BPS), and Measure of Intermittent and Constant Osteoarthritis Pain (ICOAP). Arth Care Res. 2011;63 Suppl 11:S240–52. 10.1002/acr.20543. PMID: 22588748. 10.1002/acr.20543 22588748

[39] Fishman GS, Moore LR (1982). A Statistical Evaluation of Multiplicative Congruential Random Number Generators with Modulus 231 1. J Am Stat Assoc.

[40] Olsen O, Albertsen K, Nielsen ML, Poulsen KB, Gron SM, Brunnberg HL (2008). Workplace restructurings in intervention studies - a challenge for design, analysis and interpretation. BMC Med Res Methodol.

[41] Work environment and health in Denmark: The National Research Centre for the Working Environment; 2018 [cited 2022 04/01]. Available from: https://at.dk/media/5991/spoergeskema-2018.pdf.

[42] Skotte J, Korshøj M, Kristiansen J, Hanisch C, Holtermann A (2014). Detection of physical activity types using triaxial accelerometers. J Phys Act Health.

[43] Kuorinka I, Jonsson B, Kilbom A, Vinterberg H, Biering-Sørensen F, Andersson G (1987). Standardised Nordic questionnaires for the analysis of musculoskeletal symptoms. Appl Ergon.

[44] Ware Jr. JE, Sherbourne CD (1992). The MOS 36-item short-form health survey (SF-36). I. Conceptual framework and item selection. Med Care.

[45] Stevens ML, Crowley P, Garde AH, Mortensen OS, Nygård C-H, Holtermann A (2019). Validation of a Short-Form Version of the Danish Need for Recovery Scale against the Full Scale. Int J Environ Res Public Health.

[46] Strøyer J, Essendrop M, Jensen LD, Warming S, Avlund K, Schibye B (2007). Validity and reliability of self-assessed physical fitness using visual analogue scales. Percept Mot Skills.

[47] Clausen T, Madsen IE, Christensen KB, Bjorner JB, Poulsen OM, Maltesen T (2019). The Danish Psychosocial Work Environment Questionnaire (DPQ): Development, content, reliability and validity. Scand J Work Environ Health.

[48] Kessler RC, Barber C, Beck A, Berglund P, Cleary PD, McKenas D (2003). The World Health Organization Health and Work Performance Questionnaire (HPQ). J Occup Environ Med.

[49] Ilmarinen J. The work ability index (WAI). Occup Med. 2007;57(2):160. 10.1093/occmed/kqm008.

[50] Karlsson ML, Bergström G, Björklund C, Hagberg J, Jensen I (2013). Measuring production loss due to health and work environment problems: construct validity and implications. J Occup Environ Med.

[51] Hernán MA, Robins JM (2017). Per-Protocol Analyses of Pragmatic Trials. N Engl J Med.

[52] Moher D, Hopewell S, Schulz K, Montori V, Gøtzsche P, Devereaux P (2010). CONSORT 2010 Explanation and Elaboration: updated guidelines for reporting parralel group randomised trials. J Clin Epidemiol.

