# Supplementary figures and images for: Designing industrial work to be ‘just right’ to promote health - a study protocol for a goldilocks work intervention
Source: BMC Public Health. 2022 Feb 23;22:381. doi: 10.1186/s12889-022-12643-w (PMC8867863; doi:10.1186/s12889-022-12643-w)

Appendices

Written informed consent form


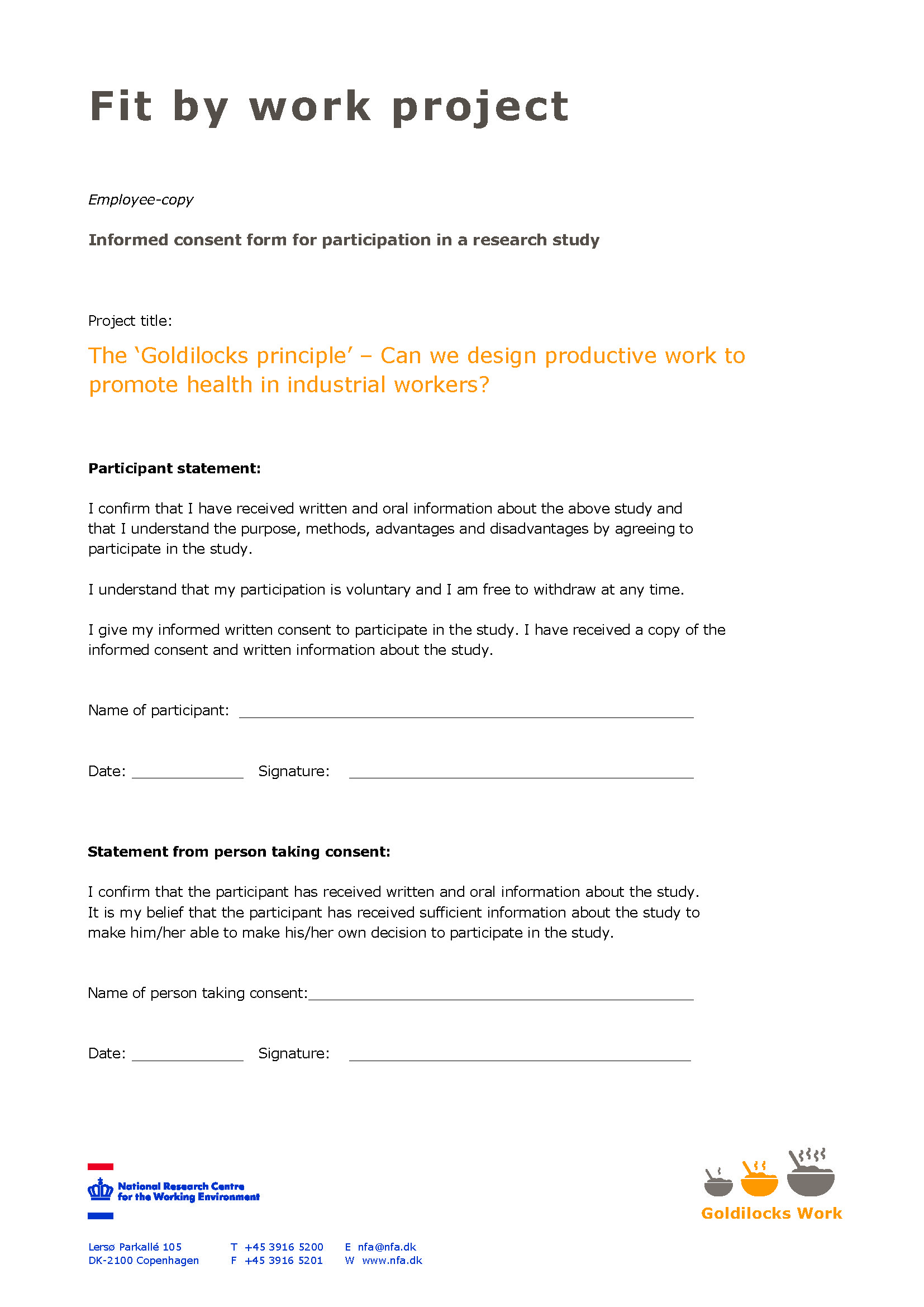

Supplement: Supplementary file 1 — Additional file 1: Appendices. Written informed consent form. [file 12889_2022_12643_MOESM1_ESM.docx]
